# Bats Increase the Number of Cultivable Airborne Fungi in the “Nietoperek” Bat Reserve in Western Poland

**DOI:** 10.1007/s00248-016-0763-3

**Published:** 2016-04-15

**Authors:** Tomasz Kokurewicz, Rafał Ogórek, Wojciech Pusz, Krzysztof Matkowski

**Affiliations:** Department of Vertebrate Ecology and Paleontology, Institute of Biology, Wrocław University of Environmental and Life Sciences, Kożuchowska 5b, 51-631 Wrocław, Poland; Department of Genetics, Institute of Genetics and Microbiology, University of Wrocław, Przybyszewskiego 63/77, 51-148 Wrocław, Poland; Department of Plant Protection, Wrocław University of Environmental and Life Sciences, pl. Grunwaldzki 24a, 50-363 Wrocław, Poland

**Keywords:** Airborne fungi, Bats, “Nietoperek” bat reserve, *Pseudogymnoascus destructans*

## Abstract

The “Nietoperek” bat reserve located in Western Poland is one of the largest bat hibernation sites in the European Union with nearly 38,000 bats from 12 species. Nietoperek is part of a built underground fortification system from WWII. The aims of the study were (1) to determine the fungal species composition and changes during hibernation season in relation to bat number and microclimatic conditions and (2) evaluate the potential threat of fungi for bat assemblages and humans visiting the complex. Airborne fungi were collected in the beginning, middle and end of hibernation period (9 November 2013 and 17 January and 15 March 2014) in 12 study sites, one outside and 11 inside the complex. Ambient temperature (*T*_a_) and relative humidity (RH) were measured by the use of data loggers, and species composition of bats was recorded from the study sites. The collision method (Air Ideal 3P) sampler was used to detect 34 species of airborne fungi including *Pseudogymnoascus destructans* (Pd). The density of airborne fungi isolated from the outdoor air samples varied from 102 to 242 CFU/1 m^3^ of air and from 12 to 1198 CFU in the underground air samples. There was a positive relationship between number of bats and the concentration of fungi. The concentration of airborne fungi increased with the increase of bats number. Analysis of other possible ways of spore transport to the underground indicated that the number of bats was the primary factor determining the number of fungal spores in that hibernation site. Microclimatic conditions where Pd was found (median 8.7 °C, min-max 6.1–9.9 °C and 100 %, min-max 77.5–100.0 %) were preferred by hibernating *Myotis myotis* and *Myotis daubentonii*; therefore, these species are most probably especially prone to infection by this fungi species. The spores of fungi found in the underground can be pathogenic for humans and animals, especially for immunocompromised persons, even though their concentrations did not exceed limits and norms established as dangerous for human health. In addition, we showed for the first time that the air in bats hibernation sites can be a reservoir of Pd. Therefore, further study in other underground environments and wintering bats is necessary to find out more about the potential threat of airborne fungi to bats and public health.

## Introduction

Specific microclimatic conditions in underground sites used by bats for hibernation are one of the most inhospitable habitats for microbial life due to low temperatures and scarcity of organic matter [1–4]. Stable and low temperature ca. 10 °C is generally the only factor beneficial to development of psychrophilic microorganisms, e.g. for *Pseudogymnoascus destructans* (Pd) having optimal growth temperatures between 12.5 and 15.8 °C and the upper critical temperature between 19.0 and 19.8 °C [5]. Therefore, fungi are commonly observed growing on organic matter in any underground environments but are present regularly as spores, carried in by water, air currents, animals (bats, arthropods) and humans [1, 6, 7]. According to Ogórek et al. [8, 9], the external environment and air currents have the main influence on number and species composition of airborne fungi in underground spaces. Most of the fungi are found in the twilight zone and in places situated near the entrances or ventilation shafts [2–4, 10].

Johnson et al. [11] isolated 42 fungi species from the wing membranes of hibernating bats, 73 % of species belonging to class *Ascomycota*, 14 % to *Basidiomycota* and 13 % to *Zygomycota*. However, number, species composition and seasonal dynamics of airborne fungal associated with bats are still poorly known, especially in Europe. Many previous studies evidenced that other fungi, especially from *Aspergillus* and *Penicillium* group producing large numbers of spores, could be harmful for both animal and human health by causing mycosis and mycotoxicosis, allergies, dysfunction of the immune system and infections of internal organs (e.g. bone marrow, intestines, kidneys) as well as inflammations of the retina, lungs, peritoneum, and urethral system [12–14].

Currently, most studies are focused on Pd, the pathogenic fungus causing white-nose syndrome (WNS), described as a widespread, epizootic disease affecting hibernating bats. WNS started in the north-eastern USA and Canada, is continuously spreading south and west and is associated with an unprecedented bat mortality exceeding 30–99 % [15–19]. However, recent investigations confirmed the presence of this fungus, but without associated mass mortality, in fifteen countries: Austria, Belgium, Switzerland, Czech Republic, Germany, Denmark, Estonia, France, Hungary, Netherlands, Poland, Romania, Slovakia, Turkey and Ukraine [18, 20–22].

Underground corridors of the central sector of the Międzyrzecz Fortified Front (MFF) in Western Poland form the eighth largest bat hibernation site in the European Union, protected as Natura 2000 site “Nietoperek” and are closed to visitors during hibernation period, i.e. from 15th October to 15th April. The maximal bat number, 38,594 individuals of 10 species, with the predomination of species from genus *Myotis* was recorded there in January 2015 [23]. In Europe, eight species of *Myotis* have been observed being colonised by Pd: *Myotis myotis*, *Myotis blythii*, *Myotis mystacinus*, *Myotis daubentonii*, *Myotis dasycneme*, *Myotis nattereri*, *Myotis bechsteinii* and *Myotis brandtii* [18], and all of them, except *M. blythii* and *M. brandtii*, occur in large numbers in the Nietoperek. Such high density of hibernating bats could put them in danger of fungal infections, especially by Pd. Until now, the only case of presence of that fungal pathogen from Poland was recorded in 2010 on *M. myotis* in the southern part of the country [18]. Additionally, a study made in Nietoperek in January 2010–2012 by sampling of fungi from bats’ muzzles using Scotch tape and microscopic examining of the spores (Kokurewicz T., Wibbelt G., Rachwald A., Schofield H., Glover A., Duverge L., Haddow J., Whitby D., Hargreaves D., pers. observations) did not prove the presence of Pd. Since 1999 in Nietoperek, the total number of bats has been constantly increasing. An exception to this trend is the number of Daubenton’s bat (*M. daubentonii*) [24–26]. In the years 1999–2013, a statistically significant population decline of that species was recorded [27]. It is still unclear if these trends could be caused by lack of Pd in that hibernation site, or that the pathogenic fungus is present there but has no impact on bat population numbers, possibly apart from Daubenton’s bat.

The aims of the study were (1) to determine the fungal species composition and changes during hibernation season in relation to bat number and microclimatic conditions and (2) evaluate the potential threat of fungi for bat assemblages and humans visiting the complex.

## Material and Methods

### Study Area

**Fig. 1:**
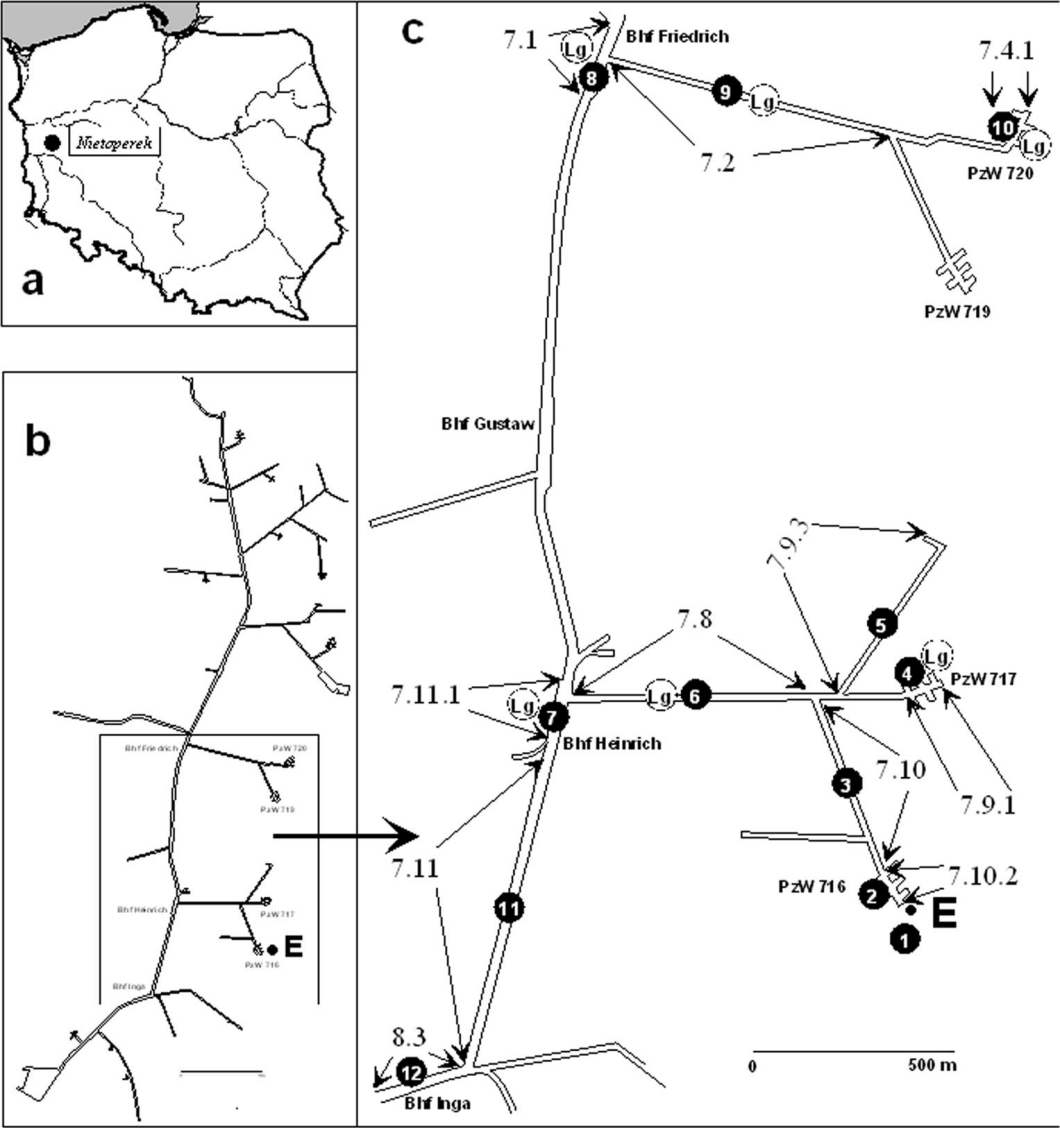
“Nietoperek” bat reserve in Western Poland. **a** Geographic location. **b** The outline of the underground fortification system. **c** Study sites and sections (7.1–8.3) where bats assemblages were recorded in November 2013 and January and March 2014. *E* entrance, *from 1 to 12* fungal sampling points (*1* outside the underground system, *from 2 to 12* inside the underground), *Lg* places were temperature/relative humidity data loggers were installed, *PzW* panzerwerk, bunker, *Bhf* “Bahnhof,” railway station

The study was done in the underground corridors of the central sector of the Międzyrzecz Fortified Front (MFF) (52°25’ N, 15°32’ E) in Western Poland (Fig. 1). The MFF was built by the Germans in the 1930s during World War II and consists of above ground bunkers connected by underground railway tunnels of total length of ca. 32 km located ca. 20–30 m underground [28]. In November 2007, the underground system with the surrounding surface area of 7377.37 ha became protected as Natura 2000 site Nietoperek (area code PLH080003). The targets of protection in MFF are four bats species, i.e. *M. myotis*, *Barbastella barbastellus*, *M. dasycneme* and *M. bechsteinii* mentioned in Annex II of the EC Directive 92/43/EEC of 21 May 1992 on the Conservation of Natural Habitats and of Wild Fauna and Flora (http://ec.europa.eu/environment/nature/legislation/habitatsdirective/index_en.htm) and hibernating there in large numbers.

### Bat Monitoring

**Fig. 2:**
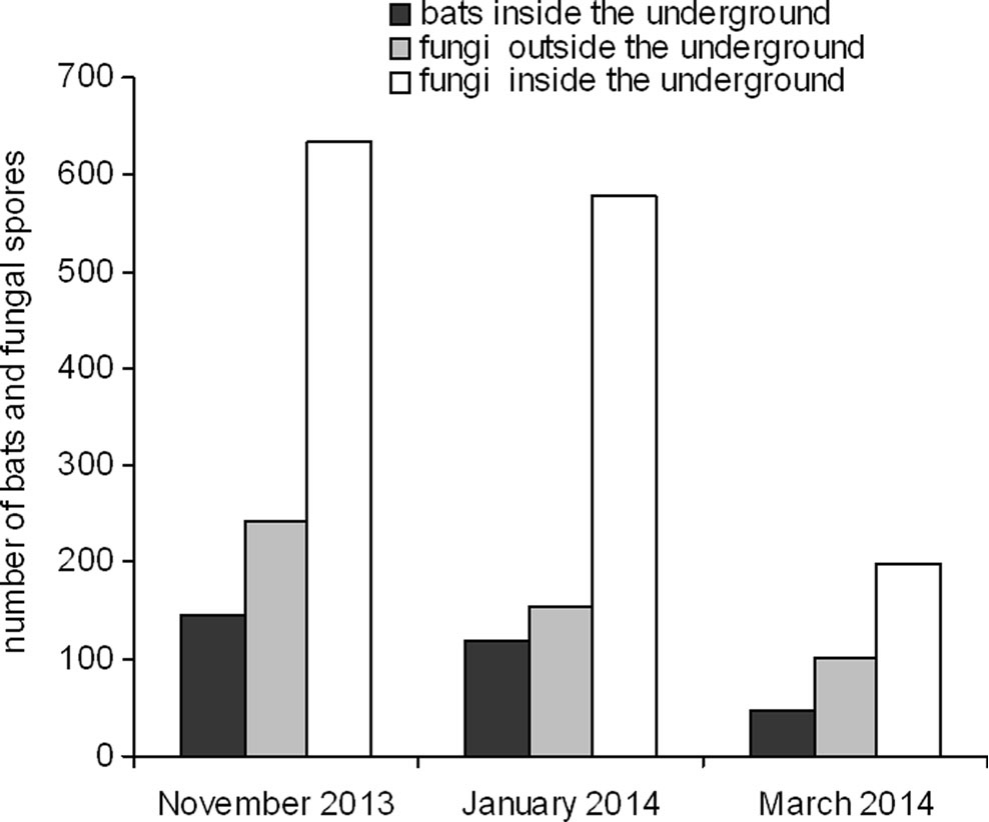
The average number of bats (individuals) and airborne fungal spores (CFU/1 m^3^ of air) recorded inside and outside the “Nietoperek” underground

For long-term monitoring of bat numbers, the underground system was divided into nine main sections [26]. The present study was undertaken in the sections 7 and 8 in the central part on the MFF (Fig. 1). Sections 7.9.1, 7.8 and 7.11.1 were available for tourists in winter, contrary to sections 7.1, 7.2 and 7.4.1 where human access was forbidden during hibernation season (Fig. 1). Bats were visually counted and identified to the species in nine sections of corridors where mycological observations were carried out. Due to legal reasons, bats were not counted in study sites 11 and 12, and consequently, those sites were excluded from analysis of relationship between bats and fungi. Because of difficulties in species identification without handling whiskered bat (*M. mystacinus*) and Brandt’s bat (*M. brandtii*), they were recorded as *M. mystacinus* and *M. brandtii* group (Fig. 2, Table 1). The observations were made under the licence issued by Nature Conservancy Management in Gorzów Wielkopolski.

**Table 1:** The numbers of fungi (CFU/1 m^3^—colony-forming unit per 1 m^3^ of air) of *Pseudogymnoascus destructans* (Pd) and numbers of individuals of bat species occurring in “Nietoperek” underground in November 2013 (XI), January 2014 (I) and March 2014 (III)

Pd or bad species/observation periods	Study site number/Pd or bat number									Total number of individuals
	2	3	4	5	6	7	8	9	10	
*Pseudogymnoascus destructans*										
XI					1		9	17	2	29
I		1		9			3	17	3	33 (+1 in study site 12)
III										0
*Myotis myotis*										
XI	14	9		45	16	29	275	387	102	877
I	25	2		19	8	5	160	383	16	618
III	20			3	1		69	196	2	291
*Myotis daubentonii*										
XI	20	9	5	45	8	15	58	40	27	227
I	27		2	63	1	6	50	23	17	189
III	7			17		1	18	12	10	65
*Myotis nattereri*										
XI	1		1	3		4	15	6	10	40
I	17		11	28		7	41	12	9	125
III								1	3	4
*Myotis mystacinus/Myotis brandtii*										
XI						1				1
*Myotis bechsteinii*										
XI							1			1
I							1			1
*Myotis dasycneme*										
XI							2			2
I							1			1
III							1			1
*Barbastella barbastellus*										
I						1				1
*Plecotus auritus*										
XI			2	1		1	7	3	1	15
I			1	1	1	1	10	5	1	20
*Eptesicus serotinus*										
III							1			1
*Not determined to species (Indet.)*										
XI				3			1			4
I							1			1
III							3		1	4

### Microclimatic Parameter Measurement

Ambient temperature (*T*_a_) and relative humidity (RH) were collected during the observation period (November–March) by use of automatic data loggers (Dallas IButton, Model DS1923, Dallas Semiconductors, TX, USA), accuracy: ±0.5 °C, ±5 % RH. Six data loggers were placed in study sites where fungi were sampled, with the programmed sampling interval of 8 h (Fig. 1, Table 2). In the places where the loggers were not installed, the air temperature and relative humidity were measured during the study period by use of thermohygrometer LB-522 (LAB-EL), accuracy: ±0.1 °C, ±2 % RH.

**Table 2:** Study sites and an average of all three sampling times microclimatic conditions in studied parts of “Nietoperek” underground

Study site number	Name	Section	temperature (°C) χ, LQ-UQ, min-max, *n*	Relative humidity (%) χ, LQ-UQ, min-max, *n*
2.	PzW 716	7.10.2	9.1 9.1–9.1 8.9–9.3 9	71.1 70.0–71.1 68.0–72.0 9
3.	Corridor from PzW 716 to PzW 717	7.10	9.5 9.4–9.6 9.0–9.8 9	75.5 74.0–76.0 72.0–78.0 9
4.	PzW 717	7.9.1	9.8 9.7–9.9 9.6–9.9 381	75.2 67.8–79.5 47.1–95.0 381
5.	Blind corridor “Gallery”	7.9.3	9.1 9.1–9.2 8.9–9.3 9	75.3 74.0–76.0 72.0–78.0 9
6.	From “Gallery” to Bhf Heinrich	7.8	9.4 9.3–9.5 9.2–10.4 381	92.0 89.2–100.0 84.4–100.0 381
7.	Bhf Heinrich	7.11.1	9.6 9.4–9.9 9.2–10.4 381	76.2 67.0–78.4 48.1–92.6 381
8.	Bhf Friedrich	7.1	8.1 7.7–8.4 7.4–8.7 381	100.0 99.2–100.0 68.7–100.0 381
9.	From Bhf Friedrich to PzW 720	7.2	8.7 8.2–9.4 6.1–9.9 381	100.0 97.0–100.0 77.5–100.0 381
10.	PzW 720	7.4.1	8.6 7.7–9.1 5.4–9.6 381	100.0 98.8–100.0 67.6–100.0 381
11.	GDR from Bhf Heinrich to Bhf Inga	7.11	9.1 9.0–9.2 8.8–9.4 9	98.1 97.0–98,1 95.0–100.0 9
12.	Bhf Inga	8.3	9.2 9.1–9.3 8.9–9.5 9	85.1 85.0–86.0 83.0–88.0 9

*PzW (panzerwerk)* bunker, *Bhf (Bahnhof)* railway station, *GDR* main road in the underground from north to south, χ median, *LQ-UQ* lower and upper quartile, *min-max* minimal and maximal values, *n* sample size

### Mycological Evaluation of the Air and Fungal Identification

The samples were collected in the beginning, middle and end of hibernation period, i.e. on the 9th November 2013, 17th January 2014 and 15th March 2014, in 12 study sites, one outside near the entrance and 11 inside the underground fortification system (Fig. 1, Table 3). The collision method with Air Ideal 3P sampler (bioMérieux) and Potato Dextrose Agar (PDA, Biocorp) medium were used for the isolation of fungi from the air. The air sampler was programmed for air sample volumes of 50, 100 and 150 L. Measurement in every study site was performed in six replicates for each volume. The sampler was positioned 1.5 m above the level of the floor. The incubation of the cultures was carried out at 15 °C and room temperature (25 °C) for 4–42 days in darkness.

**Table 3:** The total and average number of airborne fungi isolated in “Nietoperek” underground (CFU/1 m^3^ of air) in November 2013

Fungal species	Study site number												Means^a^
	1	2	3	4	5	6	7	8	9	10	11	12	
*Absidia glauca*	0	1	0	0	0	1	0	17	64	0	0	7	8.2
*Alternaria alternata* complex	17	87	0	16	8	2	43	98	50	66	14	43	38.8
*Alternaria botrytis*	0	0	0	0	3	0	6	0	0	13	0	1	2.1
*Aspergillus* sp. section *Nigri*	6	8	0	5	0	3	17	43	0	1	0	2	7.2
*Aspergillus* sp. section *Flavi*	0	0	2	0	7	0	11	0	13	0	0	0	3.0
*Aspergillus* sp. section *Fumigati*	0	9	0	0	3	0	0	34	46	6	0	0	8.9
*Aspergillus* sp. 1 section *Circumdati*	0	47	12	0	0	8	17	0	0	0	0	12	8.7
*Aspergillus* sp. 2 section *Circumdati*	3	12	43	0	0	0	23	115	0	0	0	0	17.5
*Candida albicans*	0	0	0	0	0	0	3	0	32	82	0	1	10.7
*Chaetomium globosum* complex	0	13	0	0	0	0	0	11	52	2	0	0	7.1
*Cladosporium cladosporioides* complex	78	354	211	149	34	27	189	265	188	103	124	169	164.8
*Cladosporium herbarum* complex	5	0	9	0	3	1	32	0	17	10	0	17	8.1
*Clonostachys rosea*	8	11	0	0	1	3	0	89	18	0	2	0	11.3
*Fusarium oxysporum* complex	28	0	6	0	0	0	0	0	0	0	0	0	0.5
*Mucor flavus*	0	0	0	0	0	0	6	4	0	0	0	13	2.1
*Mucor hiemalis*	9	84	0	7	11	5	3	67	56	32	8	3	25.1
*Mucor luteus*	0	0	0	2	0	0	0	8	13	1	8	0	2.9
*Mucor racemosus*	0	0	18	0	0	0	0	20	43	22	23	28	14.0
*Paecilomyces fumosoroseus*	0	0	0	0	3	0	23	5	43	13	0	7	8.5
*Paecilomyces variotii* complex	0	0	32	0	0	11	0	53	68	3	0	13	16.4
*Penicillium* sp. 1 section *Chrysogena*	43	203	115	296	73	52	285	0	138	102	98	0	123.8
*Penicillium* sp. 2 section *Chrysogena*	17	11	4	0	0	22	3	0	18	13	3	0	6.7
*Penicillium* sp. 3 section *Chrysogena*	7	16	0	0	4	0	7	0	31	0	19	1	7.1
*Penicillium* sp. 1 section *Citrina*	3	0	0	0	4	18	0	113	0	80	0	14	20.8
*Penicillium* sp. 2 section *Citrina*	0	20	0	0	16	2	0	6	0	17	27	85	15.7
*Penicillium* sp. section *Exilicaulis*	4	0	18	0	0	2	32	15	0	0	0	0	6.1
*Phoma* sp.	0	0	0	6	0	0	0	23	6	0	0	0	3.2
*Pseudogymnoascus destructans*	0	0	0	0	0	1	0	9	17	2	0	0	2.6
*Rhizopus stolonifer*	0	48	0	2	17	5	0	0	82	0	0	1	14.1
*Rhodotorula rubra*	0	0	0	0	0	2	19	17	36	7	0	0	7.4
*Sarocladium strictum*	2	17	4	44	11	5	0	17	6	88	0	0	17.5
*Trichoderma harzianum*	11	52	0	66	0	1	62	23	159	0	0	0	33.0
Non-sporulating white colonies	1	15	0	2	0	16	0	13	2	0	0	1	4.5
Non-sporulating black colonies	0	19	0	0	2	11	0	18	3	0	1	0	4.9
In total	242	1027	474	595	200	198	781	1083	1201	663	327	418	633.4

*1* outside the underground, *2–12* inside the underground

^a^Means of CFU/1 cm^3^ of air for study side from 2 to 12 (inside the underground)

Generally, specific identification of the sampled fungi was performed using macro- and microscopic observations, namely the morphology of hyphae, conidia and sporangia, of the colonies that grew on PDA. Additionally, for macro-morphological observations of fungal, species of the genus *Penicillium* and *Aspergillus* were used the following mediums: PDA, Czapek-Dox agar (1.2 % agar, Biocorp), Czapek Yeast Autolysate agar (CYA), Malt Extract agar (MEA), Yeast Extract Sucrose agar (YES), Dichloran 18 % Glycerol agar (DG18), Oatmeal agar (OA) and Creatine agar (CREA) [29]. The isolates of *Penicillium* and *Aspergillus* were inoculated on each plate of each medium and incubated at 25 °C (additional CYA plates were incubated at 30, 33 and 37 °C) in the dark, for 7 days. For micromorphological observations, all fungi, microscopic mounts, were made in lactic acid from PDA, or MEA and DG18 colonies. Alcohol was used to remove excess conidia and prevent air bubbles. The fungi were identified using by diagnostic keys and monographs [30–38] for the filamentous fungi and diagnostic key and monographs [39, 40] for the yeast-like fungi.

### Statistical Analysis

Normality of distribution of ambient temperature (*T*_a_) and relative humidity (RH) was tested by the use of Shapiro-Wilk’s *W*-test. For parameters with distribution significantly different from normal (*P* > 0.05), the medians (χ) lower quartile (LQ) and upper quartile (UQ) were calculated, and the minimum and maximum values (range) and sample size (*n*) were presented. The Pearson (*r*) correlation coefficient and regression equation (least squares, model I) was calculated to investigate the relationships between number of bats and number of fungi spores in the nine study sites (2–10) situated in the undergrounds, where bats were present. Calculations were performed by the use of Statistica ver. 9.0 (StatSoft, Inc. (2009). STATISTICA data analysis software system, 9.0. www.statsoft.com).

## Results

The presence of 9 bat taxa and 34 of airborne fungi (32 filamentous fungi and 2 yeasts) was recorded in the total study period (Tables 3, 4 and 5). The number of bats was highest in November (1167 individuals of 7 taxa), slightly reduced in January (956 individuals of 7 taxa) and dropped to the lowest number in March (366 individuals of 5 taxa)—Table 1. The largest numbers of fungi species (34) as well as the highest number of spores were observed in the underground in November (628.5 CFU/1 m^3^ of air); in January, species number remained the same; but number of spores slightly declined (579.4 CFU/1 m^3^ of air), while in March 2014, a strong decline down to 12 taxa and 199.4 CFU/1 m^3^ air was observed (Tables 3, 4 and 5, Fig. 2).

**Table 4:** The total and average number of airborne fungi isolated in “Nietoperek” underground (CFU/1 m^3^ of air) in January 2014

Fungal species	Study site number												Means^a^
	1	2	3	4	5	6	7	8	9	10	11	12	
*Absidia glauca*	0	0	0	0	9	0	0	9	7	0	0	0	2.3
*Alternaria alternata* complex	5	11	4	23	8	5	37	51	32	32	8	14	20.5
*Alternaria botrytis*	12	0	0	2	3	11	0	19	0	7	0	0	3.8
*Aspergillus* sp. section *Nigri*	0	2	0	17	0	0	17	4	2	0	0	0	3.8
*Aspergillus* sp. section *Flavi*	0	0	0	0	7	0	11	32	13	0	0	0	5.7
*Aspergillus* sp. section *Fumigati*	0	0	2	0	3	0	0	7	46	0	0	7	5.9
*Aspergillus* sp. 1 section *Circumdati*	0	86	7	0	74	16	17	0	0	24	0	1	20.5
*Aspergillus* sp. 2 section *Circumdati*	15	2	9	4	0	0	23	105	0	3	0	0	13.3
*Candida albicans*	0	2	2	0	17	0	3	11	32	41	0	1	9.9
*Chaetomium globosum* complex	0	0	11	0	0	0	0	4	52	6	0	0	6.6
*Cladosporium cladosporioides* complex	17	246	164	58	274	43	113	235	188	136	104	108	151.7
*Cladosporium herbarum* complex	0	2	0	0	3	1	10	6	17	0	0	7	4.2
*Clonostachys rosea*	0	6	0	0	76	3	0	45	18	0	2	0	13.6
*Fusarium oxysporum* complex	0	9	0	0	0	0	7	0	0	6	0	3	2.3
*Mucor flavus*	1	0	0	0	0	0	0	5	0	0	0	1	0.5
*Mucor hiemalis*	14	33	17	7	11	5	17	50	56	4	8	7	19.5
*Mucor luteus*	0	11	11	2	0	0	0	16	13	1	0	0	4.9
*Mucor racemosus*	0	16	2	0	0	0	0	7	43	19	0	0	7.9
*Peacilomyces fumoroseus*	1	11	0	0	3	0	23	21	43	16	0	5	11.1
*Peacilomyces variotii* complex	0	0	7	0	0	11	0	30	68	0	0	8	11.3
*Penicillium* sp. 1 section *Chrysogena*	38	254	43	214	132	95	285	87	138	152	75	82	141.5
*Penicillium* sp. 2 section *Chrysogena*	7	15	6	0	67	17	7	4	31	0	19	11	16.1
*Penicillium* sp. 3 section *Chrysogena*	0	1	0	0	0	5	3	29	18	1	3	4	5.8
*Penicillium* sp. 1 section *Citrina*	3	8	1	0	4	0	0	32	0	32	0	2	7.2
*Penicillium* sp. 2 section *Citrina*	0	20	0	0	16	2	0	27	0	66	27	43	18.3
*Penicillium* sp. section *Exilicaulis*	32	0	8	0	0	2	32	0	0	14	0	19	6.8
*Phoma* sp.	0	0	0	6	12	0	0	0	6	3	0	0	2.5
*Pseudogymnoascus destructans*	0	0	1	0	9	0	0	3	17	3	0	1	3.1
*Rhizopus stolonifer*	0	5	0	2	2	5	0	0	82	1	0	0	8.8
*Rhodotorula rubra*	0	0	1	0	17	2	13	5	36	2	0	8	7.6
*Sarocladium strictum*	6	5	0	7	11	0	9	22	14	30	0	0	8.9
*Trichoderma harzianum*	2	2	0	66	16	1	29	0	159	13	0	0	26.0
Non-sporulating white colonies	2	5	0	2	0	16	0	0	2	0	10	1	3.3
Non-sporulating black colonies	0	12	1	0	2	11	0	3	3	4	0	9	4.1
In total	155	764	297	410	776	251	656	869	1136	616	256	342	579.4

*1* outside the underground, *2–12* inside the underground

^a^Means of CFU/1 cm^3^ of air for study side from 2 to 12 (inside the underground)

**Table 5:** The total and average number of airborne fungi isolated in “Nietoperek” underground (CFU/1 m^3^ of air) in March 2014

Fungal species	Study site number												Means^a^
	1	2	3	4	5	6	7	8	9	10	11	12	
*Alternaria alternata* complex	0	0	0	0	0	0	5	0	0	0	0	0	0.5
*Alternaria botrytis*	0	0	0	5	0	40	0	0	0	5	0	0	4.5
*Cladosporium cladosporioides* complex	0	30	4	4	0	11	4	0	21	2	20	0	8.7
*Cladosporium herbarum* complex	30	10	20	0	0	0	30	5	5	5	0	20	8.6
*Mucor flavus*	0	0	0	0	0	0	0	5	0	0	0	0	0.5
*Mucor hiemalis*	0	0	10	0	0	0	0	0	0	0	0	0	0.9
*Peacilomyces fumoroseus*	0	0	0	0	10	0	0	0	0	0	0	0	0.9
*Penicillium* sp. 1 section *Chrysogena*	40	45	60	125	0	100	90	110	225	60	120	165	100.0
*Penicillium* sp. 2 section *Chrysogena*	2	0	0	0	0	0	0	0	0	0	0	0	0.0
*Penicillium* sp. section *Citrina*	0	2	5	0	0	0	0	0	0	0	0	0	0.6
*Penicillium* sp. section *Exilicaulis*	0	85	25	35	0	135	115	105	70	0	105	95	70.0
*Phoma* sp.	5	0	0	0	0	0	0	0	0	0	0	0	0.0
*Trichoderma harzianum*	0	0	0	1	0	0	0	0	0	0	30	2	3.0
Non-sporulating white colonies	25	5	5	0	2	0	0	0	0	0	0	0	1.1
In total	102	177	129	170	12	286	244	225	321	72	275	282	199.4

*1* outside the underground, *2–12* inside the underground

^a^Means of CFU/1 cm^3^ of air for study side from 2 to 12 (inside the underground)

**Fig. 3:**
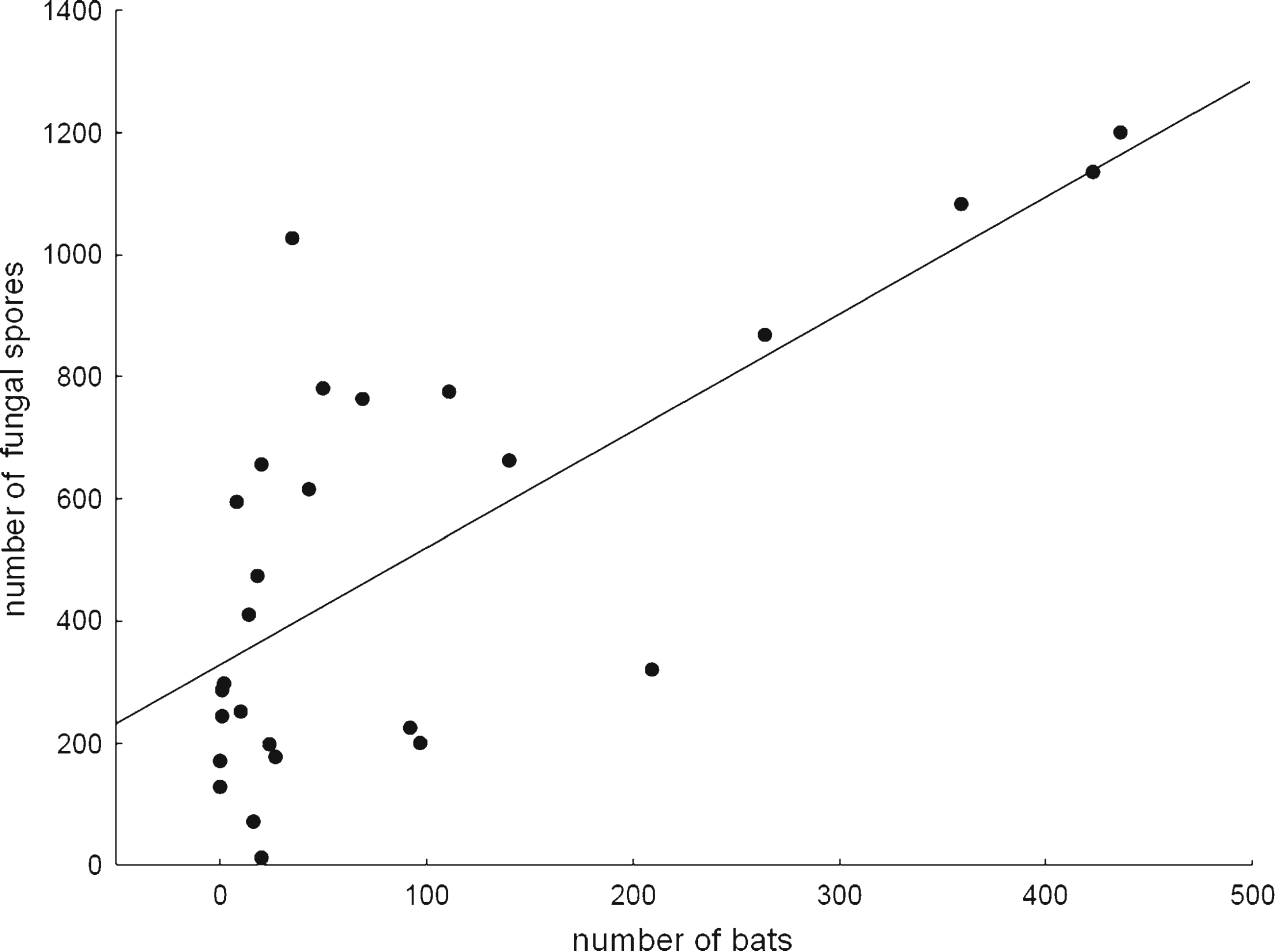
Relationship between number of bats and airborne fungal spores (CFU/1 m^3^ of air) in the nine study sites (2–10) situated inside the “Nietoperek” underground. The Pearson correlation coefficient, statistical significance and regression equation are *r* = 0.71, *d.f.* = 1,25, *P* < 0.0001; *y* = 328.29 + 1.916*x*

The highest numbers of bats during all inspections were recorded in study site 9, where the mouse-eared bat (*M. myotis*) was the most numerous species in all three observation periods exceeding 387 individuals in November and 196 in March (Fig. 1, Table 1). In that location, during all inspections, we also observed the largest number of fungal spores reaching the highest number in November (1198 CFU/1 m^3^ of air), remaining high in January (1136 CFU/1 m^3^ of air) and significantly declining in March down to 321 CFU/1 m^3^ of air (Tables 3, 4 and 5, Fig. 3). In the above ground reference study site (Fig. 1), much lower number of fungi species (20 during all three inspections) and number of spores from 242 to 155 in November and January, and to 102 CFU/1 m^3^ of air in March, were recorded (Tables 3, 4 and 5).

We found an association between number of bats and the count of fungal spores—Fig. 2. The concentration of airborne fungi increased with the increase of bats number in the underground study sites. Highly statistically significant positive correlation was found between those two variables (*r* = 0.71, *d.f.* = 1,25, *P* < 0.0001; *y* = 328.29 + 1.916*x*)—Fig. 3. *Cladosporium cladosporioides* complex was the most frequently isolated fungi from samples taken both outside and inside the underground in November; in January, it was found only in samples taken inside corridors. *Penicillium* sp. 1 from section *Chrysogena* was most frequently isolated from both places in March and from the outside samples in January (Tables 3, 4 and 5).

The spores of Pd were recorded only in November (29 CFU/1 m^3^ of air) and January (34 CFU/1 m^3^ of air) in study sites 3, 5, 6, 8–10 and 12, but most of them (17 CFU/1 m^3^ of air) were found in study site 9 (Fig. 1), with the largest numbers of bats, mainly mouse-eared bat (*M. myotis*), were recorded during all inspections (Table 1). The median temperature and relative humidity in study site 9 were 8.7 °C (min-max 6.1–9.9 °C) and 100 % (min-max 77.5–100.0 %)—Table 2. In that part of the underground tourist movement is forbidden in winter, contrary to the sections 7.9.1, 7.8 and 7.11.1, it could be assumed that only bats are responsible for both transport and high number of spores of Pd in that section of tunnels.

Fungi from genera *Aspergillus* and *Penicillium* were the most numerous species group of airborne fungi isolated during all study periods. *Aspergillus* spp. were not observed in March but constituted from 7.1 to 8.5 % of all spores recorded in November and January, while *Penicillium* spp. constituted 28.5 % in November, 34.2 % in January and 83.6 % in March of all recorded CFU/1 m^3^ (Tables 3, 4 and 5).

## Discussion

According to the results of previous study, the most important factors affecting the survival of fungi are air temperature and humidity. Because of the presence of fungal spores in bioaerosols, their concentrations are the result of complex interactions between biological and environmental factors. Due to the dynamic nature of the atmosphere, the individual importance of each factor is hard to assess, especially in specific conditions observed underground [41]. However, according to many reports, the most important factors determining occurrence of fungal spores in underground spaces are airflow, the availability of organic matter and the conditions prevailing in the neighbouring external environment. Generally, larger numbers of fungi are isolated from air samples taken outside than inside underground sites [2, 3, 8–10, 42]. Contrary to these results, during our study, most of the spores were isolated from the air samples taken inside the underground corridors. The results of previous study indicated that number and species composition of fungi was positively correlated with number of tourists and bats visiting underground [3, 43–48], which was confirmed by a positive relationship between number of bats and number of fungi spores in the air we found in our study site. In section 9, where the largest number of spores was recorded, the air movement, which could potentially transport the spores, runs from the entrance situated in section 7.4.1, whereas study plot 10 was situated the rear part of the underground corridors (Figs. 1 and 3). In study plot 10, the number of spores was lower than in section 9 (Fig. 3); moreover, in that part of the underground, tourist movement is forbidden in winter. Additionally, study plot 9 is situated ca. 750 m from the entrance and probably due to that no insects and other animals were recorded there during our study. Based on that, we can assume that bats may be one of the reasons for the increase in the number of spores in section 9 through the production of guano and the transport of spores from the external environment. According to Ogórek et al. [49], bat guano is a very good substrate for the development and survival of fungi inside underground sites, and it can also be a reservoir of fungi harmful to bats and humans.

Another aim of our study was to evaluate the risk of high concentrations of airborne fungi for human health. Some of the experts propose 5000 CFU of fungi in 1 m^3^ of air as acceptable [50]. According to the Polish norm PN-89/Z-04111/03 [51], the air can be not contaminated if it contains no more than 3000 CFU of fungal spores in 1 m^3^, but on the other hand, the World Health Organization suggests that the concentration of airborne fungi as high as 1500 CFU in 1 m^3^ air is acceptable, but only if it is a mixture of species [52]. The overall mean concentration of CFU found during our study was from 102 to 628.5 CFU/1 m^3^ in the air outside the underground and from 12 to 1198 inside of it. In other underground fortifications in Poland, similar concentrations of airborne fungi, e.g. from 245.5 to 1040.3 CFU (Rzeczka complex), 92–259 CFU (Osówka complex) and 25–1003 CFU (Włodarz complex), were recorded [2, 3, 9]. Summarising, the concentrations of airborne fungi in the Nietoperek bat reserve did not exceed official limits and norms for prevention of a health risk to humans.

In study site 9, where the largest number of Pd spores was detected (Figs. 1 and 3, Table 1), tourist movement does not occur in winter, which allows the assumption that only bats are responsible for both transport and growth of this fungal species in that section of the tunnels. In addition, microclimatic conditions in this area such as low temperatures and high relative humidity (8.7 °C, 100 %) are favourable for the growth of Pd [17], and they are also preferred by hibernating mouse-eared bats (*M. myotis*) [53] and Daubenton’s bats (*M. daubentonii*) [54], occurred in Nietoperek in large numbers (Table 1).

The lack of spores of Pd in March is contradictory to the results of a previous study indicating the highest numbers of that species towards the end of hibernation season, i.e. in March and April [18]. Our study showed that in November and January, when the number of bats and spores of Pd associated with them were high, it was possible to detect the presence of this species in Nietoperek, contrary to the low bat and spore numbers in March, which probably made it more difficult to detect the presence of that fungal species.

The study made in the Nietoperek underground during bat censuses in January 2010–2012 using a standard protocol of sampling of fungi from muzzles of bats by Scotch tape, followed by examination of spores under the microscope (Kokurewicz T., Wibbelt G., Rachwald A., Schofield H., Glover A., Duverge L., Haddow J., Whitby D., Hargreaves D., pers. observations) did not prove the presence of Pd. An additional factor which should be considered when applying this procedure is the sensitivity of hibernating bats to tactile disturbance [55] leading to additional energy loss [56]. Based on that future study directed at the influence of Pd on hibernating bat populations, we can recommend the method described above and tested during our study. Due to the easily repeatable sampling procedure and especially the low risk of harm to bats, we would recommend it as the first step, to be followed by more detailed investigation aimed at potential influence of Pd on hibernating bat populations, especially Daubenton’s bat (*M. daubentonii*), a species declining in number in many localities in Europe.

Our study is the first aero-mycological evaluation being done in a large bat hibernation site aimed at describing the fungal species composition and its changes during hibernation season by using culture-based analysis and collision method. In this method, the suction force ensures adherence of all the fungal propagules to the surface of a suitable culture medium. Furthermore, we can accurately determine their number allocated to each volume of the air. This method is very fast, and a large number of samples can be easily taken during a short time period. Moreover, small air samplers, such as the Air Ideal 3P, are useful in difficult study conditions such as underground sites [57].

Currently, literature reported that Pd is transmitted with direct contact between bats or with contaminated environment bats such as soil and sediment [18, 19, 58]. Probably, this fungus can be also mechanically transmitted by adhesive spores and mycelium fragments on the body of ectoparasites such as spinturnicid mites [59]. We showed for the first time that the air can be also a reservoir of Pd, and it is likely that the fungus can be transmitted through the air. However, we do not know (1) the length of time the structure of this fungus retains its potential for propagation and to be infectious in the air and also (2) how many spores in the air are necessary to infect a bat. Therefore, further study of Pd in the air is necessary to find answers to the above questions. It seems that it will be particularly difficult to determine the relationship between an infection and the number of spores in the air, because the result of the infection depends on determinants of the pathogen, host(s) and the environment. Any changes in these determinants may trigger shifts in the complex host-pathogen system [60].

*Penicillium* from section *Chrysogena*, e.g. *Penicillium chrysogenum* and *C. cladosporioides* complex were the fungi species most frequently isolated from air samples taken from aboveground and underground study sites. Fungi of the genus *Penicillium* are cosmopolitan species able to produce spores in low temperatures observed in the underground, and these have been identified as important allergens in the indoor environment and as a rare causative agent of opportunistic mycosis in humans [61–67]. Fungi of the genus *Cladosporium* are also a cosmopolitan and common endophytic fungi [68, 69]. Additionally, studies of atmospheric air of various regions in Europe show that spores of *Cladosporium* spp. represent ca. 80 % of all the caught spores, with the peak season for sporulation from June to September when several thousand spores are produced per cubic metre of air [70, 71]. *Cladosporium* are very commonly isolated airborne fungi from the external and internal air of caves and other underground sites [2, 4, 8, 42].

The presence of toxic and allergenic fungi positively correlated in number with the number of bats should be considered when planning tourist movement in the underground spaces occupied by bats, such as Nietoperek bat reserve and many others. Based on our results, we suggest that veterinary examination of bats, and medical examination of bat workers and underground tourist guides, is necessary to find out more about the potential threat to bats and to public health also in other underground environments and wintering bats.

## Conclusions

Our study is the first aero-mycological evaluation of a large bat hibernation site aimed at describing the fungal species composition and its changes during the hibernation season. Generally, the density of airborne fungi isolated from the underground air was higher than in the outdoor air samples but did not exceed official limits and norms established as dangerous for human health. We detected a positive relationship between number of bats and number of fungal spores underground. The large number of bats and the lack of tourists in the study sites with largest numbers of spores indicate that the presence of these animals appears to be the primary factor determining number and species composition of fungi in the underground sites. *C. cladosporioides* complex was the fungal species most frequently isolated from the air samples taken both outside and inside the underground system in November, but only from inside in January. *Penicillium* sp. 1 from section *Chrysogena* was most frequently isolated from both places in March and from the outside in January. Microclimatic condition where Pd was found was preferred by hibernating *M. myotis* and *M. daubentonii*; therefore, these species are most probably especially prone to infection by this fungi species. In addition, the most frequently detected fungi genera were *Aspergillus* and *Penicillium* that can produce mycotoxins and cause infections. The collision method involving the Air Ideal 3P sampler and collecting spores on Petri dishes with appropriate solidified culture medium proved to be a good way to detect the fungi harmful to bats such as Pd. Moreover, sampling of airborne fungi is non-invasive, in contrast to direct examination of bats, and may be conducted at a time when bats are absent in hibernacula. Therefore, we recommend the use of this method as the first step in a mycological study of bat hibernation sites, to be followed by more detailed investigations aimed at recognising the potential influence of Pd on hibernating bat populations. The fungi species found in the underground can be pathogenic for human health and animals, especially for immunocompromised persons. In addition, we showed the first time that the air can be also a reservoir of Pd, and it is likely that the fungus can be transmitted through the air. Therefore, further study of bats and people visiting the underground environment is necessary to find out more about the potential threat to these animals as well as to public health.
